# Endometrial carcinomas with ambiguous histology often harbor *TP53* mutations

**DOI:** 10.1007/s00428-024-03912-7

**Published:** 2024-09-05

**Authors:** Ben Davidson, Karin Teien Lande, Daniel Nebdal, Anne Jorunn Nesbakken, Arild Holth, Kristina Lindemann, Ane Gerda Zahl Eriksson, Therese Sørlie

**Affiliations:** 1 Department of Pathology, Norwegian Radium Hospital, Oslo University Hospital, Montebello, N-0310 Oslo, Norway; 2 Faculty of Medicine, Institute of Clinical Medicine, University of Oslo, N-0316 Oslo, Norway; 3 Department of Cancer Genetics, Institute for Cancer Research, Norwegian Radium Hospital, Oslo University Hospital, Montebello, N-0310 Oslo, Norway; 4 Section for Gynecologic Oncology, Division of Surgical Oncology, Norwegian Radium Hospital, Oslo University Hospital, Oslo, Norway

**Keywords:** Ambiguous endometrial carcinoma, Whole-exome sequencing, TP53, Survival

## Abstract

The objective of the present study was to characterize the molecular features of endometrial carcinomas with ambiguous histology. Eighteen carcinomas that could not be conclusively typed based on morphology and immunohistochemistry underwent analysis of mismatch repair (MMR) status, microsatellite status, and whole-exome sequencing. None of the tumors had pathogenic *POLE* mutation. Twelve tumors (67%) were microsatellite stable, and 6 (33%) had microsatellite instability. Fourteen tumors (78%) harbored *TP53* mutations, and 2 (11%) had mutations in MMR genes. Eleven carcinomas (61%) were classified as copy number high and 7 (39%) as MSI-hypermutated, the latter including 3 tumors with *TP53* mutation who concomitantly had MSI or mutation in a MMR gene. Other mutations that were found in > 1 tumor affected *MUC16* (7 tumors), *PIK3CA* (6 tumors), *PPP2R1A* (6 tumors), *ARID1A* (5 tumors), *PTEN* (5 tumors), *FAT1* (4 tumors), *FAT4* (3 tumors), *BRCA2* (2 tumors), *ERBB2* (2 tumors), *FBXW7* (2 tumors), *MET* (2 tumors), *MTOR* (2 tumors), *JAK1* (2 tumors), and *CSMD3* (2 tumors). At the last follow-up (median = 68.6 months), 8 patients had no evidence of disease, 1 patient was alive with disease, 8 patients were dead of disease, and 1 patient died of other cause. In conclusion, based on this series, the molecular landscape of endometrial carcinomas with ambiguous histology is dominated by *TP53* mutations and the absence of *POLE* mutations, with heterogeneous molecular profile with respect to other genes. A high proportion of these tumors is clinically aggressive.

**Supplementary Information:**

The online version contains supplementary material available at 10.1007/s00428-024-03912-7.

## Introduction

Endometrial cancer, consisting predominantly of endometrial carcinoma (EC), is the 6th most common cancer in women globally. In 2020, it accounted for 417,367 new cancer diagnoses and 97,370 deaths [1]. In developed countries, it is the 4th common cancer in women, and its incidence is on the rise, primarily due to an increase in obesity [2].

EC histotypes include endometrial endometrioid carcinoma (EEC), serous carcinoma (SC), clear cell carcinoma (CCC), mucinous carcinoma (MC), mesonephric-like carcinoma, neuroendocrine carcinoma, dedifferentiated and undifferentiated carcinoma, and mixed tumors and tumors with hybrid phenotype and genetic profile. Additionally, carcinosarcomas (CS) are now regarded as biphasic malignancies derived from EC, most commonly SC [3].

The TCGA study identified 4 molecularly and clinically distinct EC groups, consisting of *POLE* ultramutated, microsatellite instability hypermutated, copy-number low, and copy-number high tumors [4]. Follow-up studies have demonstrated that a surrogate panel consisting of immunohistochemistry (IHC) for p53 and mismatch repair (MMR) proteins, combined with *POLE* mutations analysis, can reliably assign the majority of EC into one of the 4 abovementioned groups [reviewed in 5].

While the majority of EC can be reliably classified based on morphology and IHC, some cases with ambiguous features remain challenging. The latter include tumors harboring *POLE* mutation and tumors with deficient MMR (dMMR) from patients with Lynch syndrome, but tumors that do not belong to either of these groups are not rare.

Studies that have specifically focused on EC with ambiguous features have been few to date. Analysis of 13 cases by Espinosa et a. showed heterogenous IHC and molecular features in this group [6].

In the process of reviewing a large series of high-grade EC from our archives, with the objective of identifying grade 3 EEC (G3EEC) and SC, we identified 18 carcinomas that could not unequivocally be assigned to a histologic entity. The present study focuses on the genetic analysis of these tumors.

## Material and methods

### Patients and specimens

The study material consisted of a series of 18 hysterectomy specimens, submitted to the Departments of Pathology at the Norwegian Radium Hospital and Ullevål University Hospital (currently both part of Oslo University Hospital) for routine diagnostic purposes during the period 2007–2019. Tumors from recent years were diagnosed by experienced gyn-pathologists, whereas some of the earlier cases were signed out also by pathologists with less specialized training. Overall, 10 tumors were diagnosed as adenocarcinomas, NOS, 2 as SC, 2 as G3EEC, 2 as mixed SC-EEC, 1 as CS, and 1 as undifferentiated carcinoma.

The number of slides containing tumor in the hysterectomy specimens ranged from 1 to 10, with 3–4 tumor slides available in the majority of cases (12/18; 67%). In the 3 specimens for which < 3 slides were available, the reason was either the presence of a small tumor in a polyp (1 case, 2 slides containing tumor) or removal of most tumor tissue in the pre-operative curettage (1 case with 1 slide, 1 with 2 slides).

Specimens were independently reviewed by two experienced pathologists with sub-specialty in gynecologic pathology (BD and AJN) as part of a larger review of high-grade carcinomas focusing on grade 3 EEC (G3EEC) and SC. Cases were subsequently discussed in a consensus meeting. All cases underwent staining for p53 and MMR proteins, and additional IHC was performed as regarded necessary, using various combinations of the antibodies listed in Table 1. Lack of agreement with the original diagnosis or between the two observers after re-evaluation of morphology and IHC staining led to inclusion in the present study.

**Table 1:** Antibodies

Antibody	Manufacturer	Catalog #	Clone	Host	Dilution	Antigen retrieval
AE1/AE3	Dako (Glostrup, Denmark)	M3515	AE1/AE3	Mouse	1:250	HpH pH 9
AFP	Dako	A0008	polyclonal	Rabbit	1:100	None
AMACR (p504s)	Dako	M3616	13H4	Rabbit	1:100	HpH pH 9
ARID 1A	Sigma-Aldrich (St. Louis MO)	HPA005456	polyclonal	Rabbit	1:400	LpH pH 6
E-Cadherin	Zymed Laboratories (Seattle WA)	13–1700	HECD-1	Mouse	1:3000	HpH pH 9
Calretinin	Dako	M7245	DAK-Calret1	Mouse	1:100	HpH pH 9
CD 10	Novocastra (Newcastle upon Tyne)	NCL-CD10-270	56C6	Mouse	1:50	HpH pH 9
CEA	Dako	M7072	II-7	Mouse	1:200	HpH pH 9
Chromogranin A	Millipore (Burlington MA)	MAB5268	LK2H10	Mouse	1:30000	LpH pH 6
CK7	Dako	M7018	OV-TL 12/30	Mouse	1:300	HpH pH 9
CK8	Abcam (Cambridge, UK)	AB193094	EP1628Y	Rabbit	1:1500	LpH pH 6
EMA	Dako	M0613	E29	Mouse	1:200	HpH pH 9
ER	Novocastra	MCL-ER-6F11	6F11	Mouse	1:200	HpH pH 9
GATA3	Cell Marque (Rocklin CA)	390 M-14	L50-823	Mouse	1:600	HpH pH 9
HNF1β	Atlas (Stockholm, Sweden)	HPA002083	polyclonal	Rabbit	1:300	LpH pH 6
MLH1	Dako	M3640	ES05	Mouse	1:50	LpH pH 6
MSH2	Dako	M3639	FE11	Mouse	1:50	LpH pH 6
MSH6	Dako	M3646	EP49	Mouse	1:50	LpH pH 6
Napsin A	BioGenex (Fremont CA)	MU701-5UC	IP64	Mouse	1:100	HpH pH 9
p16	NeoMarkers (Fremont CA)	MS-887-P	16P04	Mouse	1:500	LpH pH 6
p53	Santa Cruz (Santa Cruz CA)	sc-126	DO-1	Mouse	1:500	HpH pH 9
PAX8	Biosite (Solihull, UK)	10,336–1-AP	polyclonal	Rabbit	1:400	LpH pH 6
PMS2	Dako	M3647	EP51	Rabbit	1:40	LpH pH 6
PR	Novocastra	NCL-PGR	1A6	Mouse	1:300	HpH pH 9
PTEN	Dako	M3627	6H2.1	Mouse	1:100	HpH pH 9
Synaptophysin	Dako	M7315	DAK-SYNAP	Mouse	1:100	HpH pH 9
TAG-72	BioGenex	MU054-UC	B72.3	Mouse	1:1000	LpH pH 6
TTF1	Atlas	HPA003425	polyclonal	Rabbit	1:500	LpH pH 6
Vimentin	Dako	M0725	V9 (3)	Mouse	1:1600	HpH pH 9
WT-1	Dako	IR05561-2	6F-H2	Mouse	rtu	HpH pH 9

One of the pathologists (BD) selected the paraffin blocks and tumor area to be analyzed by whole-exome sequencing (WES). Minimum tumor cell content was set at 30%, and the majority of specimens contained > 50% tumor cells.

Clinicopathologic data are presented in Table 2. Study approval was given by the South-Eastern Norway Committee for Medical Research Ethics.

**Table 2:** Clinicopathologic parameters

Case	Age	BMI	FIGO stage^*a*^	LVSI	LN resected	Omentum resection	Recurrence	TTR	OS	Status
1	56	29.8	II	Yes	Pelvic	Yes	Lung	9	25	DOD
2	76	25.8	II	Yes	Pelvic + aortic	No	Lung + vagina	28	63	DOD
3	48	30.5	IA	No	Pelvic + aortic	No	No	-	169	NED
4	70	23.6	IB	Yes	Pelvic + aortic	Yes	Vagina	21	45	DOD
5	77	23.8	IA	No	Pelvic + aortic	Yes	Multiple	46	59	DOD
6	74	26	IB	No	Pelvic	No	Lung	30	51	DOD
7	79	29.3	IB	No	Pelvic + aortic	No	No	-	69	DOC
8	66	41	IA	No	Pelvic + aortic	Yes	No	-	99	NED
9	74	50.9	IA	No	SLN	No	No	-	74	NED
10	60	38.1	IA	No	SLN	No	No	-	55	NED
11	70	37.3	II	Yes	Pelvic + aortic	No	No	-	143	NED
12	69	22.5	IVB	No	Pelvic	Yes	Multiple	12	72	AWD
13	60	34.1	IA	No	Pelvic	Yes	Pelvis	23	86	NED
14	74	16.4	IA	No	Pelvic + aortic	Biopsy	No	-	85	NED
15	83	28.2	IIIC1	Yes	Pelvic	No	Multiple	19	46	DOD
16	79	26.1	IA	No	Pelvic	Yes	No	-	88	NED
17	65	36.6	IA	No	Pelvic	Yes	Pelvis + lung	29	79	DOD
18	68	29.3	IIIC2	Yes	Pelvic + aortic	Yes	Multiple	8	58	DOD

Abbreviations: *BMI*, body mass index; *LVSI*, lymphovascular space invasion; *LN*, lymph nodes; *TTR*, time to recurrence; OS, overall survival; *NA*, not available; *NED*, no evidence of disease; *AWD*, alive with disease; *DOD*, dead of disease; *DOC*, dead of other cause; *SLN*, sentinel lymph node

^*a*^ 2009 staging

### Whole-exome sequencing (WES)

#### DNA isolation

Tissue sections (two to five 10–20-µm-thick sections per patient) from formalin-fixed paraffin-embedded (FFPE) tumor and normal tissue were incubated overnight at 45 °C before deparaffinization in limonene and rehydration in a series of graded alcohol solutions. Areas of tumor cells were carefully scraped off the tumor tissue slides with a scalpel using adjacent H&E-stained sections as guides and transferred to lysis buffer in Eppendorf Safe-Lock tubes. From the normal tissue slides, the entire area was included. DNA and RNA were then extracted on a Qiacube (Qiagen, Hilden, Germany), using the Qiagen AllPrep DNA/RNA FFPE protocol, with buffer EB replacing buffer ATE as elution buffer. The isolated DNA was quantified using the Qubit dsDNA HS Assay Kit (Thermo Fisher, Waltham MA, USA).

#### Genomic analysis

Tumor and normal DNA were sequenced at the OUH Genomics Core Facility using Twist Human Core Exome enrichment (Twist Bioscience, South San Francisco CA, USA). Libraries were sequenced paired-end 2 × 150 bp on the NovaSeq6000 System (Illumina, San Diego CA, USA). The average coverage was 369.9 for the tumor samples and 157.2 for the normal samples.

#### Variant calling

Variant calling and analysis were performed with Illumina Dragen Bio-IT v. 3.9. The normal samples were first separately analyzed with the germline pipeline to identify potential single nucleotide (SNVs) and structural (SVs) variants. Tumor and normal samples were then analyzed with the tumor-normal analysis pipeline to identify somatic SNVs and SVs. The human reference genome GRCh38 (patch p12) (obtained from the UCSC database) was used for mapping and alignment. SNV and SV calling was performed with Twist Exome BED target file (target BED padding 250).

#### Variant annotation and filtration

The open-source software package Personal Cancer Genome Reporter (PCGR) [7] was used for somatic variant annotation. For each individual sample, a report summarizing the detected SNVs and SVs, in addition to computed tumor mutational burden (TMB), microsatellite instability (MSI) status, and mutational signatures, was generated.

Vcf files were used as input using settings “*tumor-control*, uterus samples.” Tumor reading depth (tdb) was set as > 1, and tumor allele frequency (taf) was set as > 0. SNVs with known relevance to endometrial carcinoma were extracted and filtered first for coding mutations, then for tumor reading depth ≥ 30 and tumor allele frequency ≥ 0.05. Occasional mutations with allele frequencies close to 0.5 or 1 were ignored, assuming these represent germline variations.

#### Mutational signatures

COSMIC single base substitution (SBS) mutational signatures [8] were generated for each tumor sample by the PCGR workflow [7], given that the total number of SNVs in the exome data exceeded 200. The mutational signature reconstruction was limited to the subset of SBS signatures relevant to the uterus category. An accuracy of fitting was calculated that reflects how well the mutation profile can be reconstructed based on the available signatures.

Mutational signatures were reported as the most dominant signature for a given tumor. Of note, contributions and fitting of the signatures varied among the samples (37–82%, 75–96%), and the contribution of the most dominant signature and second most dominant signature may be similar.

## Results

**Fig. 1:**
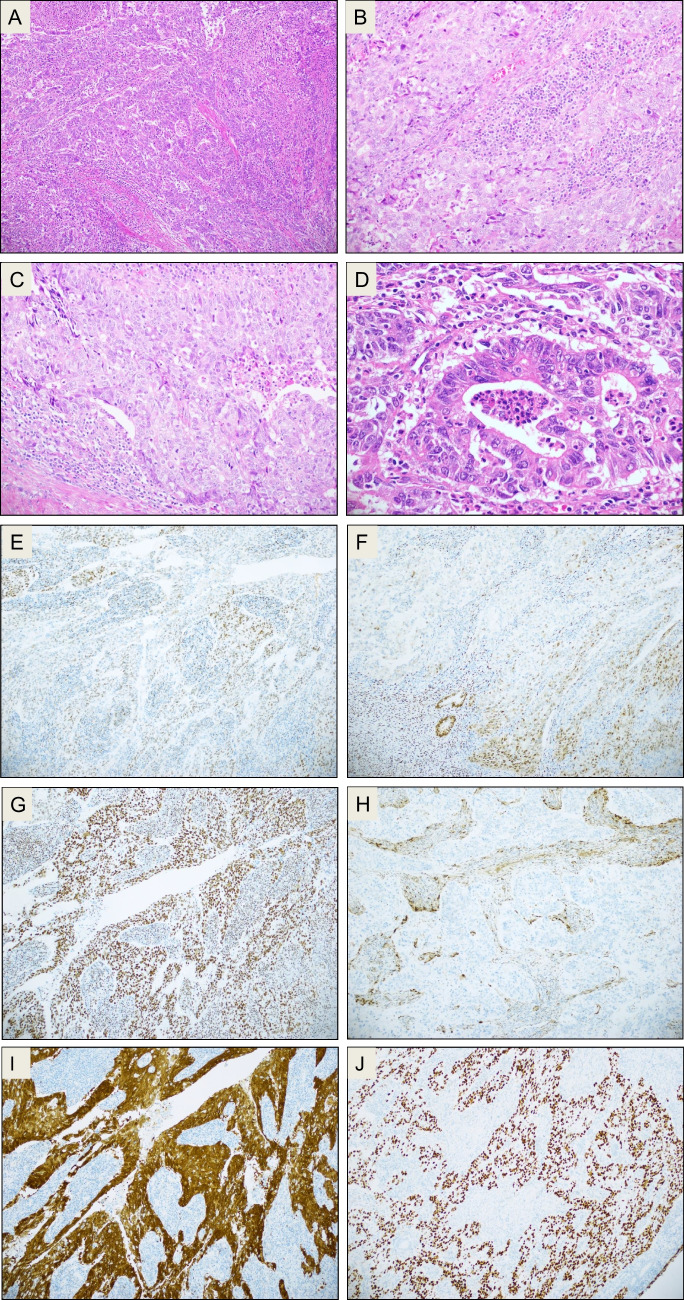
Case # 8. **A**–**D** Morphology, H&E staining: invasive adenocarcinoma with high-grade nuclear features growing predominantly with a solid pattern, with a minor component showing glandular/acinar pattern. The tumor is infiltrated by a large number of mature lymphocytes. **E**–**J** Immunostaining. The tumor is only focally positive for ER (**E**) and PR (**F**). ARID1A is retained (**G**), PTEN is lost (**H**). p16 stains with block-positivity (**I**), p53 is aberrant/mutation-type (diffusely positive; **J**)

**Fig. 2:**
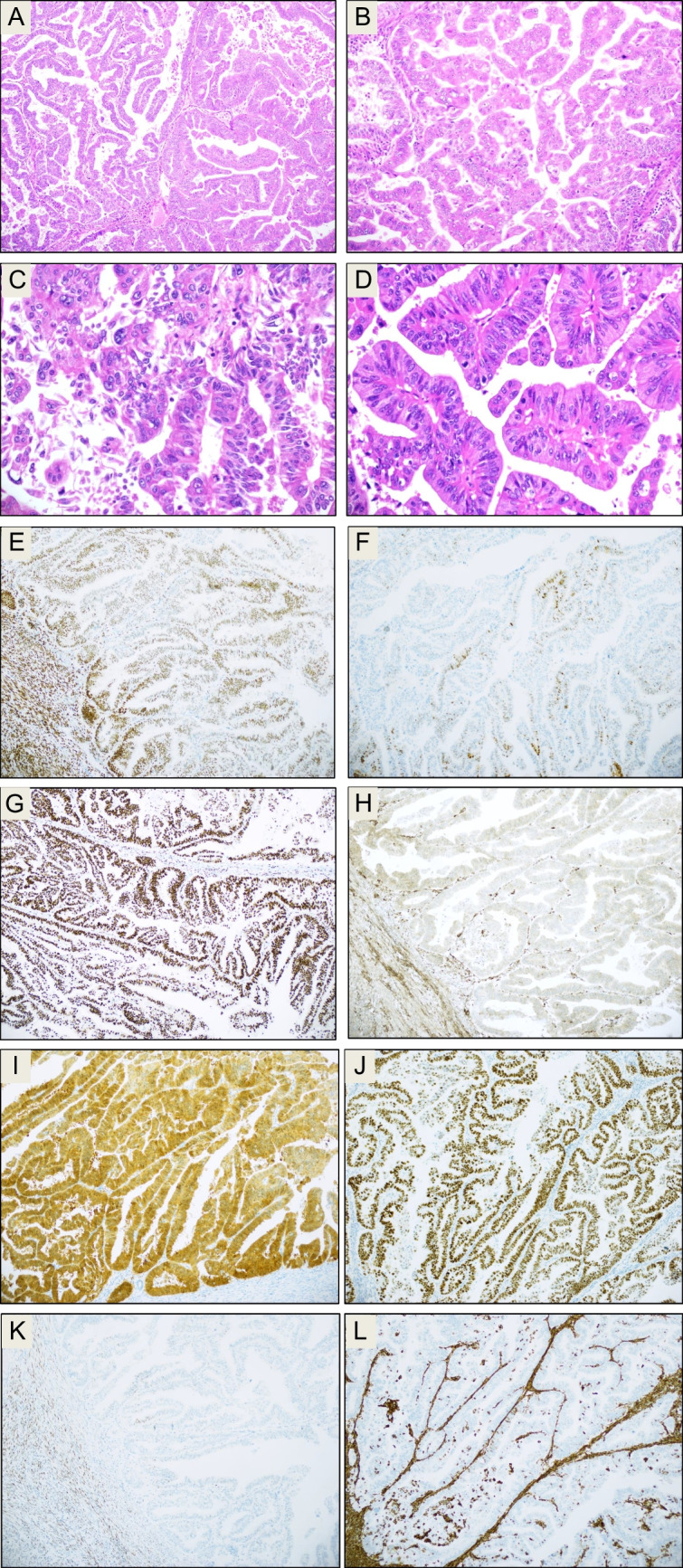
Case # 16. **A**–**D** Morphology, H&E staining: invasive adenocarcinoma growing with confluent glandular/acinar and trabecular pattern. The tumor consists of high columnar cells with high-grade nuclear features and strongly eosinophilic cytoplasm, with brisk mitotic activity. The tumor is infiltrated by some mature lymphocytes, though less densely than the case in Fig. 1. **E**–**L** Immunostaining. The tumor is partly positive for ER (**E**), but only focally positive for PR (**F**). ARID1A is retained (**G**), PTEN is lost (**H**). p16 stains with block-positivity (**I**), p53 is aberrant/mutation-type (diffusely positive; **J**). The tumor is negative for WT1 (**K**) and vimentin (**L**)

Two representative cases (#8 and #16 in Table 2) of this series of 18 EC are shown in Figs. 1 and 2. Tumors tended to be morphologically homogenous, with the exception of one specimen in which part of the tumor had endometrioid features and the other part was serous-like, though IHC failed to highlight this difference.

Fourteen tumors (78%) had aberrant (mutation-type) p53 staining patterns by IHC, and 4 (22%) had wild-type patterns. Loss of PMS2 or MSH6 occurred in 2 tumors (11%) each, with no overlap. Twelve tumors (67%) were microsatellite stable (MS), and 6 (33%) had microsatellite instability (MSI). Results are summarized in Table 3.

**Table 3:** p53, MMR, and MS status

Case	p53	MSH6	PMS2	MS status
1	Aberrant	Lost	Retained	MSI high
2	Aberrant	Retained	Retained	MSI high
3	Wild type	Lost	Retained	MSI high
4	Aberrant	Retained	Retained	MSS
5	Aberrant	Retained	Retained	MSS
6	Wild type	Retained	Retained	MSS
7	Wild type	Retained	Lost	MSI high
8	Aberrant	Retained	Retained	MSS
9	Aberrant	Retained	Retained	MSS
10	Aberrant	Retained	Retained	MSS
11	Aberrant	Retained	Retained	MSS
12	Aberrant	Retained	Retained	MSS
13	Aberrant	Retained	Retained	MSS
14	Aberrant	Retained	Retained	MSS
15	Aberrant	Retained	Retained	MSS
16	Aberrant	Retained	Retained	MSI high
17	Aberrant	Retained	Retained	MSS
18	Wild type	Retained	Lost	MSI high

Abbreviations: *MMR*, mismatch repair; *MSI*, microsatellite unstable; *MSS*, microsatellite stable

^*a*^All aberrant p53 with the diffusely positive pattern

In exome sequencing, none of the tumors had pathogenic *POLE* mutation. Fourteen tumors (78%) harbored *TP53* mutations, and 2 (11%) had mutations in MMR genes. Eleven carcinomas (61%) were classified as copy number high and 7 (39%) as MSI-hypermutated.

Other mutations that were found in > 1 tumor affected *MUC16* (7 tumors), *PIK3CA* (6 tumors), *PPP2R1A* (6 tumors), *ARID1A* (5 tumors), *PTEN* (5 tumors), *FAT1* (4 tumors), *FAT4* (3 tumors), *BRCA2* (2 tumors), *ERBB2* (2 tumors), *FBXW7* (2 tumors), *MET* (2 tumors), *MTOR* (2 tumors), *JAK1* (2 tumors), and *CSMD3* (2 tumors). Data are summarized in Supplementary Table 1.

Median follow-up was 69 months (range 25–169 months). Three patients did not receive any adjuvant therapy, while the remaining ones all received Platinol-based chemotherapy regimens.

At the last follow-up, 8 patients had no evidence of disease, 1 patient was alive with disease, 8 patients were dead of disease, and 1 patient died of other cause.

The 3 patients who did not receive chemotherapy (# 3, 14, and 16 in Table 2) did not have disease recurrence. Of note, all 3 tumors carried *TP53* mutation (Supplementary Table 1). 

## Discussion

Extensive research in recent years has compellingly shown that EC is a highly heterogenous disease with complex genetic make-up, in which molecular features are informative of the clinical behavior and thereby patient outcome. Consequently, analysis of some of these features at the protein or gene level, mainly those derived from the TCGA study, has become a diagnostic standard in many institutions in developed countries, with the potential to affect patient management [5, 9–11]. Many tumors may be thus assessed, but some defy classification. This often pertains to tumors in which morphology and IHC are equivocal. While some of these tumors, including those carrying *POLE* mutations, may have indolent behavior, it is likely that others do not.

In the present series, 7 patients died of the disease, and 1 was diagnosed with recurrence at the last follow-up, data which denote a patient group with worse outcome than the entire population diagnosed with EC. In agreement with this, *TP53* mutations, as well as aberrant p53 protein expression by IHC, were commonly found. Furthermore, while MSI and mutations in MMR genes were present in 6 and 2 cases, respectively, loss of MMR proteins by IHC was infrequent, and *POLE* pathogenic mutations were universally absent.

While the number of cases analyzed in the present study is insufficient for survival analysis, a closer look at the tumors which resulted in fatalities (# 1–2, 4–6, 15, and 17–18) does not suggest that p53 and MMR status by IHC or MS status are able to differentiate these patients from the remaining ones. The same is true for the sequencing analysis. This is particularly exemplified in the 3 patients who received no chemotherapy and experienced no disease recurrence, despite the presence of *TP53* mutation in their tumors. The latter had otherwise different genetic features.

Other mutations detected in WES analysis affected several genes known to be mutated in EC, including those more common in EEC and/or CCC (*PTEN*, *PIK3CA*, *ARID1A*), and those more commonly observed in SC or other high-risk EC, as well as CS (*PPP2R1A*, *ERBB2*, *FBXW7*, *MTOR*, *MET*). The latter are considered to be diagnostic in the differentiation between SC and EC, as well as potential therapeutic targets in high-risk EC [12–14].

*BRCA2* mutations were found in 2 tumors, of which 1 also had a *TP53* mutation. In the series by Jamieson et al., *BRCA2* mutations were found in 3% of EC with aberrant p53 expression [13]. In a recent analysis of 1625 EC of all histological types, 11 tumors carried pathogenic germline mutation in *BRCA2* [15]. In another large study, the presence of *BRCA2* mutations was more common in early-onset (< 50 years) compared to late-onset EC [16]. Treatment of these patients with poly (ADP-ribose) polymerase (PARP) inhibitors has obvious rationale.

Mutations in *FAT1* and *FAT4* were found in 4 and 3 tumors, respectively, and were not mutually exclusive. FAT genes encode FAT atypical cadherins, one of the 6 subfamilies of cadherins. Four FAT members (FAT1-4) have been identified in vertebrates. FAT proteins are transmembrane proteins involved in adhesion, migration, and proliferation, and modulate intracellular signaling via the WNT and MAPK pathways, additionally affecting epithelial-to-mesenchymal transition (EMT) [17]. Mutations in *FAT1* have been described in various cancers, including leukemias and hepatocellular carcinoma, and altered, predominantly reduced, expression of FAT1 protein has been reported in these and other cancers, the latter including head and neck, esophageal, cervical, and breast carcinoma [reviewed in 17]. Similarly, mutations in *FAT4* have been reported in various malignancies, including melanoma [18], colorectal carcinoma [19], and esophageal carcinoma [20]. Our data are in agreement with a recent study in which frequent *FAT4* mutations were found in the analysis of 9 high-risk EC, consisting of 6 SC, 1 CCC, 1 G3EEC, and 1 dedifferentiated carcinoma [21].

*JAK1* mutation was found in 2 tumors in the present study, of which one had MSI (frameshift mutation in *JAK1*) and one MSS (splice donor variant in *JAK1*). JAK1 is part of the JAK-STAT signaling pathway, which regulates the transcription of proteins involved in proliferation and cell survival. JAK family members, consisting of JAK1, JAK2, JAK3, and TYK2, are deregulated in immune response disorders, such as systemic lupus erythematosus, and in hematological cancers, and JAK inhibitors are used in the treatment of these diseases [22]. *JAK1* frameshift mutations mediate immune response evasion via inhibition of antigen presentation in microsatellite unstable EC. In a large series published by Stelloo et al., 62/181 (34%) of EC has MSI, of which 35 (22%) harbored *JAK1* mutation, though no prognostic role was found for this alteration [23]. Activation of the interferon-γ pathway is downregulated in EC carrying *JAK1* mutation [24]. *JAK1* mutations were additionally found to be significantly more common (45% vs. 4%) in EC with *MLH1* methylation compared to tumors lacking this methylation [25]. Nevertheless, in a recent phase 2 study of EC patients treated with pembrolizumab, *JAK1* mutations were not associated with resistance to this drug, as evidenced by the response in 7/10 patients with mutated tumors compared to 7/14 of patients with tumors lacking this mutation [26].

Finally, we report the finding of *CSMD3* mutation in 2 carcinomas. *CUB and sushi multiple domains 1* gene (*CSMD1*), the first gene cloned from this family, was described as a postulated adhesion molecule or transmembrane protein with a role as tumor suppressor in head and neck squamous cell carcinoma. Two other members, *CSMD2* and *CSMD3*, were subsequently identified [27]. Reduced expression of CSMD1-3 proteins was reported in colorectal carcinoma compared to patient-matched normal tissue [28]. *CSMD3* mutations were recently found in lung adenocarcinomas, with more frequent mutations in invasive compared to in situ tumors (8% vs. 3%) [29]. A previous report of *CSMD3* mutations in EC has suggested that this finding likely reflects an elevated background mutation rate rather than the accumulation of pathogenic driver mutations [30].

Given the results of the molecular analysis, we reassessed the morphology and the IHC features of the tumors and attempted to assign them to one of the TCGA tumor groups. Applying this combined knowledge, and regarding, in the absence of POLE mutations, *TP53* mutation as the determining molecular event, 14 of the carcinomas would be placed in the *TP53*-mutated category, whereas 4 would be classified as NSMP.

One obvious limitation of this study is the fact that molecular analysis was limited to a single paraffin block from each tumor. Despite the fact that tumors tended to have homogeneous morphology, the possibility that some were more heterogeneous at the genomic level cannot be entirely ruled out. Thus, for example, carcinomas initially belonging to the dMMR group may develop a clone carrying a *TP53* mutation, an event which would result in a more aggressive clinical course. Studies of intra-tumoral heterogeneity may shed more light on this issue.

In conclusion, genomic analysis of the mutation profile of EC with ambiguous pathology reveals frequent *TP53* mutations, as well as mutations in other genes related to aggressive clinical behavior, with universal absence of *POLE* pathogenic mutations. This suggests a need to regard these tumors as potentially aggressive, despite their heterogeneous clinical course. Several of the genes mutated in these tumors are relevant therapeutic targets.

## Supplementary Information

Below is the link to the electronic supplementary material.

Supplementary file1 (XLSX 19 KB)

## Data Availability

Sequencing data are available upon reasonable request.
